# Perturbed gut microbiome and fecal and serum metabolomes are associated with chronic kidney disease severity

**DOI:** 10.1186/s40168-022-01443-4

**Published:** 2023-01-09

**Authors:** Haichao Wang, Aisima Ainiwaer, Yaxiang Song, Ling Qin, Ai Peng, Hui Bao, Huanlong Qin

**Affiliations:** 1 Department of Nephrology and Rheumatology, Shanghai Tenth People’s Hospital, Tongji University School of Medicine, Shanghai, 200072 China; 2 Department of Gastrointestinal Surgery, Shanghai Tenth People’s Hospital, Tongji University School of Medicine, Shanghai, 200072 China

**Keywords:** Chronic kidney disease, Gut microbiome, Metabolome, Toxin, Oxidative stress

## Abstract

**Background:**

Chronic kidney disease (CKD) is a severe public health problem associated with a disordered gut microbiome. However, the functional alterations of microbiota and their cross talk with metabolism pathways based on disease severity remain unclear.

**Results:**

We performed metagenomics and untargeted metabolomics in a cohort of 68 patients with CKD of differing severities and 20 healthy controls to characterize the complex interplay between the gut microbiome and fecal and serum metabolites during CKD progression. We identified 26 microbial species that significantly changed in patients with CKD; 18 species changed as the disease progressed, and eight species changed only in a specific CKD group. These distinct changes in gut microbiota were accompanied by functional alterations in arginine and proline, arachidonic acid, and glutathione metabolism and ubiquinone and other terpenoid-quinone biosynthesis pathways during CKD progression. Further metabolomic analyses revealed that the distributions of toxic and pro-oxidant metabolites from these four essential metabolic pathways varied in the feces and serum as CKD progressed. Furthermore, we observed a complex co-occurrence between CKD severity-related bacteria and the characterized metabolites from the four essential metabolic pathways. Notably, *Ruminococcus bromii*, fecal hydroquinone, and serum creatinine were identified as the main contributors to the integrated network, indicating their key roles in CKD progression. Moreover, a noninvasive model including *R. bromii* and fecal hydroquinone, L-cystine, and 12-keto-tetrahydro-LTB4 levels classified the CKD severity (area under the curve [AUC]: > 0.9) and had better performance than the serum creatinine level for mild CKD (*AUC*: 0.972 vs. 0.896).

**Conclusions:**

Perturbed CKD severity-related gut microbiota may contribute to unbalanced toxic and pro-oxidant metabolism in the gut and host, accelerating CKD progression, which may be an early diagnostic and therapeutic target for CKD.

Video Abstract

**Supplementary Information:**

The online version contains supplementary material available at 10.1186/s40168-022-01443-4.

## Background

Chronic kidney disease (CKD) is a progressive disease with hidden epidemics. It considerably contributes to end-stage renal disease (ESRD), cardiovascular comorbidities, cachexia, and anemia, accounting for nearly 1.2 million deaths annually [1, 2]. Furthermore, the Asian Renal Collaboration reported that the number of adult patients with CKD increased to 434.3 million in Asia as of 2020, further escalating the already high cost of therapies and imposing a substantial economic burden on the patients and society [3]. Hence, noninvasive tests are urgently needed to identify patients at the greatest risk for advanced CKD and predisposing factors to slow disease progression.

Recently, several studies have reported a link between CKD and gut microbiota [4–6]. For example, decreased levels of *Faecalibacterium*, *Roseburia*, *Clostridium cluster IV*, *Eubacterium*, *Bifidobacterium*, and *Lactobacillaceae* and increased *Enterobacteriaceae* colonization have been reported in patients with CKD [7, 8]. In addition, rats receiving fecal microbiota transplantation (FMT) from patients with ESRD expressed more serum uremic toxins, aggravated renal fibrosis, and excessive oxidative stress than those who received FMT from healthy controls (HCs) [5]. These findings demonstrate that gut microbiota and their associated metabolites communicate with the host’s kidney via the microbiota-gut-kidney axis, making it a novel target for early diagnosis and personalized medication for slowing renal progression.

Nonetheless, functional alterations in gut microbiota and their cross talk with CKD-associated metabolism across different disease severities remain unclear. Therefore, we performed gut metagenome sequencing and fecal and serum metabolome analyses in a cohort of Chinese patients with CKD of varying severities to identify CKD severity-related changes in the microbial signatures.

## Materials and methods

### Study population and sample collection

This cross-sectional study included 68 non-dialyzed patients with CKD and 20 HCs from the Nephrology and Rheumatology Unit of Shanghai Tenth People’s Hospital affiliated with Tongji University, Shanghai, China. CKD was defined as kidney structural or functional abnormalities persisting for > 3 months. Furthermore, CKD was classified based on the estimated glomerular filtration rate (eGFR) into categories G1–G5 using the CKD Epidemiology Collaboration creatinine equation [9]. Patients with CKD were excluded if they were below the age of 18 years; had active gastrointestinal diseases, liver cirrhosis, cancer, active infection, or cardiovascular event in the past 3 months; were pregnant; or had received renal replacement therapy. Twenty age- and sex-matched HCs with normal renal and liver function who were not taking any medications were enrolled. HCs with hypertension, diabetes, obesity, and metabolic syndrome and those who were pregnant were excluded. In addition, individuals who underwent gastrointestinal surgery or took antibiotics, prebiotics, or probiotic products within 1 month were also excluded.

**Fig. 1:**
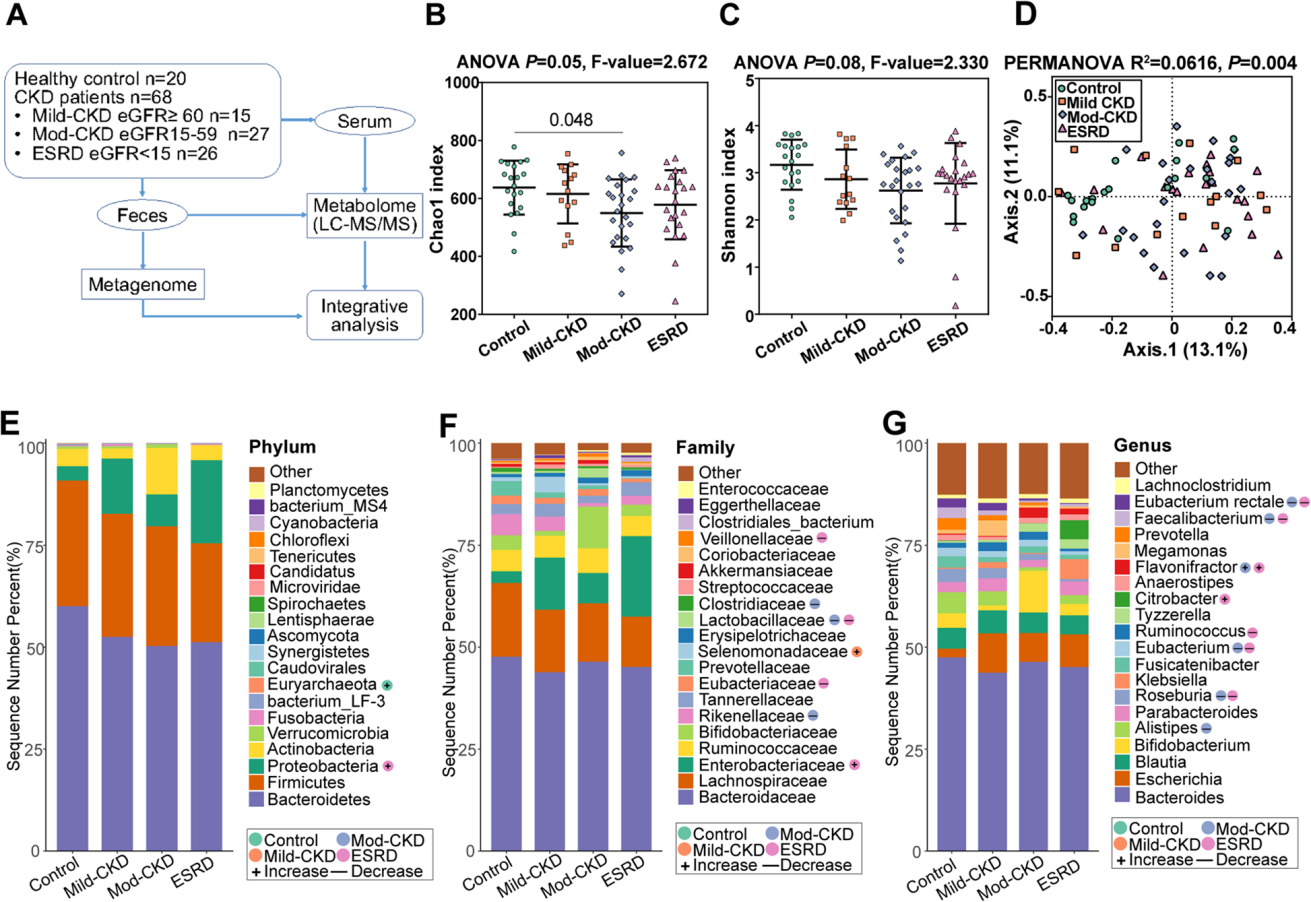
Alterations of gut microbial constructions in CKD. **A** Overview of the workflow for the metagenomic and metabolomic strategy used in this study. Comparison of *α*-diversity by **B** Chao1 and **C** Shannon indices using ANOVA in patients with chronic kidney disease (CKD) and healthy controls (HC). **D** Principal coordinate analysis (PCoA) based on the Bray–Curtis dissimilarity metric showed a significant difference in the gut microbial composition among CKD groups and HC. Statistical significance and variance of Bray–Curtis dissimilarity data were assessed using PERMANOVA. The distribution of top 20 **E** phyla, **F** family, and **G** genera detected in different phenotypic subgroups. The relative abundances of microbiota at different levels were assessed for significant elevation or depletion in each of the CKD groups with Mann–Whitney *U*-test, compared to the HC (+ , elevation with *P* < 0.05,—, depletion with *P* < 0.05). mod-CKD, moderate CKD; ESRD, end-stage renal disease

To explore disease progression, patients with CKD were categorized into the mild (*eGFR* ≥ 60 mL/min/1.73 m^2^, *n* = 15), moderate (*eGFR* 15–59 mL/min/1.73 m^2^, *n* = 27), and ESRD (*eGFR* < 15 mL/min/1.73 m^2^, *n* = 26) groups (Fig. 1A) [10, 11]. Except for completed anthropometric and laboratory indices, matched fecal and blood samples were collected for metagenomic and metabolomic analyses. Fresh stool samples were collected and stored at − 80 °C until fecal DNA extraction. Anticoagulant blood samples (5 mL) were collected on the 2nd day of the fecal collection after overnight fasting. All blood samples were clotted at room temperature for 30 min and centrifuged at 3000 × g for 10 min. Then, they were then aliquoted and stored at − 80 °C until use.

The Shanghai Tenth People’s Hospital’s Institutional Ethics Committee approved all protocols (SHYS–IEC–5.0/22K162/P01). Furthermore, we informed all patients of the study’s purpose, and they provided written informed consent. This study was performed following the 1975 Declaration of Helsinki and is registered at ClinicalTrials.gov (NCT05543291).

### Fecal DNA extraction and metagenomic sequencing

Stool sample DNA was extracted using the E.Z.N.A.® Soil DNA Kit (Omega Bio-tek, Norcross, GA, USA). DNA purity was measured using a NanoDrop 2000 Microvolume Spectrophotometer (Thermo Fisher, Waltham, MA, USA). The DNA concentrations were quantified using a TBS–380 Mini-Fluorometer (Promega, Madison, WI, USA), and the quality was determined by 1% agarose gel electrophoresis. Fecal DNA was fragmented using an M220 Focused-ultrasonicator (Covaris, LLC., Woburn, MA, USA) and screened to prepare the DNA library using a NEXTflex™ Rapid DNA-Seq Kit (Bioo Scientific, Austin, TX, USA) following the manufacturer’s instructions. All samples were paired-end sequences with a 150 bp read length on the Illumina NovaSeq platform. Then, we removed the adaptor regions, low-quality reads, and duplicated reads from the raw reads. After aligning to the human reference genome using the Burrows-Wheeler Aligner (http://bio-bwa.sourceforge.net) and filtering out human host DNA contamination, the remaining reads with acceptable quality were subjected to metagenomic analysis.

### Metabolomics preparations for fecal and serum samples

The fecal samples were thawed on ice, and then 100 mg (± 1%) of feces was added to 600 μL of 2-chlorophenylalanine (4 ppm) methanol and then vortexed for 30 s at − 20 °C. Next, the fecal samples were subjected to ultrasound at room temperature for 10 min after grinding with 100 mg glass beads for 90 s at 60 Hz. Then, the samples were centrifuged at 12,000 rpm for 10 min at 4 °C, and 300 μL of supernatant was filtered through a 0.22 μm membrane and added into the detection bottle for non-targeted metabolomics liquid chromatography-mass spectrometry (LC–MS) detection.

For the serum samples, 100 μL of the sample was mixed with 400 μL of 50% ice-cold methanol solution. After centrifugation at 12,000 rpm at 4 °C for 10 min, the supernatant was transferred into another 2 mL centrifuge tube and concentrated to dryness by vacuum. The samples were redissolved in 150 µL of 2-chlorophenylalanine (4 ppm) 80% methanol solution, and the supernatant was filtered through 0.22 µm membrane for LC–MS. A quality control sample was prepared to assess the analytical variability by mixing equal volumes (20 μL) of the supernatant from each sample.

Fecal and serum metabolic profiles were generated using a Vanquish ultra-high-performance liquid chromatography system (Thermo Fisher). We used the positive polarity mode eluents A2 (0.1% fatty acid in water) and B2 (0.1% formic acid in acetonitrile) and negative mode eluents A3 (5 nM ammonium fluoride in water) and B3 (acetonitrile). An increasing linear gradient of solvent B2/B3 (v/v) was used as follows: 0–1 min, 2%; 1–9 min, 2–50%; 9–12 min, 50–98%; 12–13.5 min, 98%; 13.5–14 min, 98–2%; and 14–20 min, 2%. The injection volume was 2 μL with a 0.25 flow rate. Electrospray ionization tandem mass spectrometry experiments were performed on a Thermo Fisher Q Exactive mass spectrometer with a spray voltage of 3.5 kV and − 2.5 kV in positive and negative modes. The source conditions were as follows: sheath gas flow rate, 30 arbitrary units; auxiliary gas flow rate, 25 arbitrary units; capillary temperature, 325 °C; full scan range, 81 to 1000 m/z; mass resolution, 7000; and normalized collision energy, 30 eV. The raw data were converted to the mzXML format using ProteoWizard software; processed for peak identification, filtration, and alignment; and normalized using the XCMS package for R software (R Core Team, Vienna, Austria). Finally, a data matrix containing the retention time, mass-to-charge ratio, and peak intensity was exported for subsequent analysis.

### Statistical analyses

#### Metagenomic data analysis

Taxonomic profiling of the gut microbiome was performed using Calypso software (V8.84; http://cgenome.net/calypso/). First, operational taxonomic unit counts were normalized by total sum normalization and corrected using cumulative sum scaling. Then, log2 transformation was applied to account for the non-normal distribution of the taxonomic count data. Taxa with an average relative abundance below 0.01% and over 95% zeroes were dropped. Samples with less than 3000 sequence reads were also removed. Alpha (α)-diversity was assessed using the Chao1 and Shannon indices. Statistical differences among all groups in α-diversity and microbial taxa were assessed by one-way analysis of variance. Beta-diversity was calculated using principal coordinate analysis (PCoA) based on the Bray–Curtis distance matrix and tested by permutational multivariate analysis of variance using the adonis function. Finally, canonical correspondence analysis (CCA), a multivariate analysis, was performed to examine the potential effects of clinical factors on CKD-related microbial signatures.

Reads originating from microbial genes were mapped to the Kyoto Encyclopedia of Genes and Genomes (KEGG) ortholog (KO). For each KO gene, the abundance of microbial genes was summed so that each component in each KO gene represented an organism. Differentially enriched KEGG pathways were identified according to the *Z*-scores of individual KOs [11, 12]. Linear discriminant effect size (LEfSe) analysis was also performed to determine the characteristic KEGG pathways of the different groups. KO genes were analyzed using the Wilcoxon rank-sum test for any of the CKD groups compared to the HC group.

**Fig. 2:**
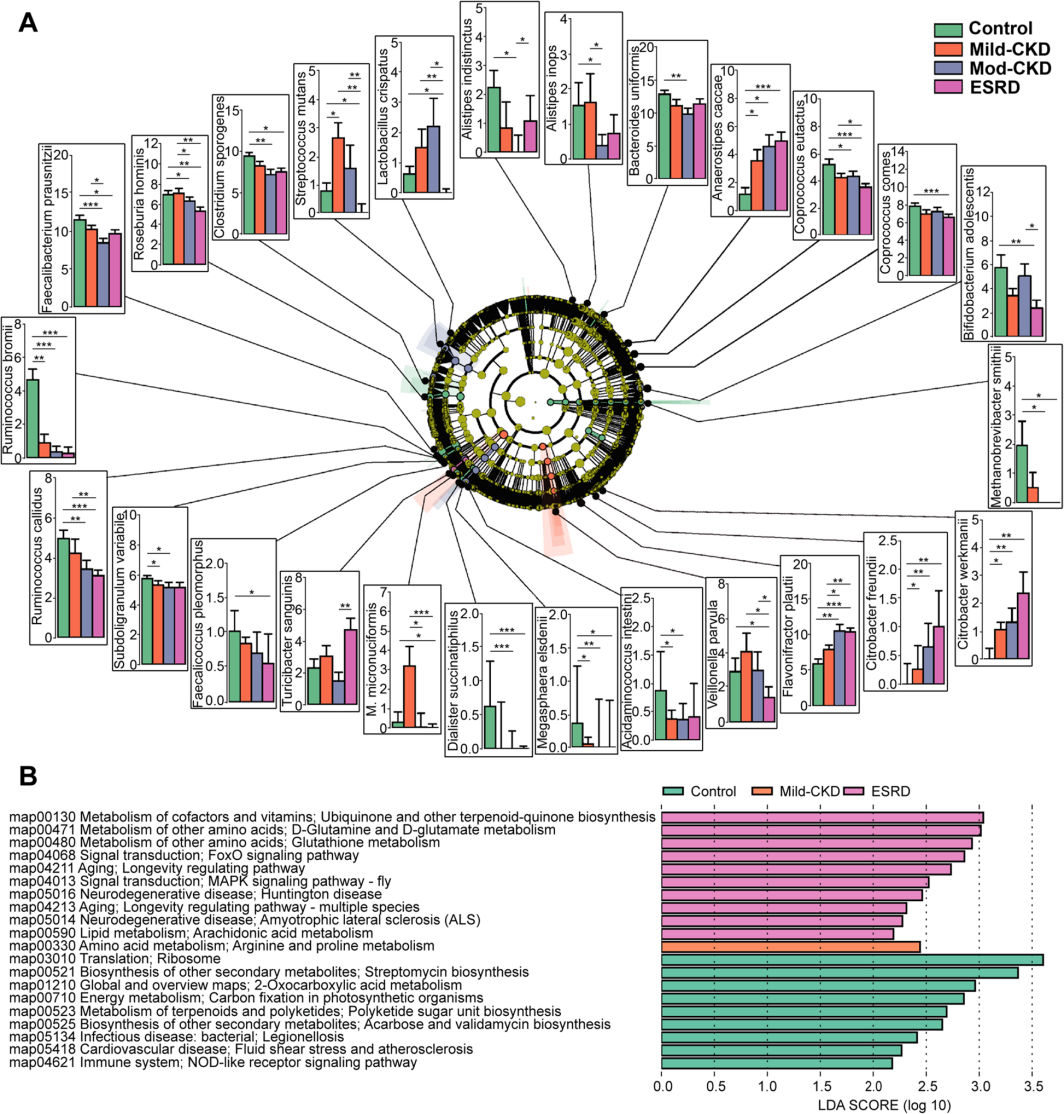
Alterations of the microbial species compositions and functions in CKD. **A** Taxonomic analysis showed 26 microbial species were changed in CKD groups compared to the healthy controls (HC). Species level was described in terms of log2 transformed abundance after total sum normalization and cumulative-sum scaling. Statistical analysis was performed by ANOVA (*P<0.05, **P<0.01, ***P<0.001). **B** Linear discriminant analysis (LDA) effect size (LEfSe) analysis defined characteristically microbial functions of different groups based on KEGG pathways (LDA score > 2). mod-CKD, moderate CKD; ESRD, end-stage renal disease; *M. mcronuciformis*, *Megasphaera micronuciformis*

#### Non-targeted metabolomics analysis

All metabolite annotations were confirmed by matching them with entries in the Human Metabolome Database (http://www.hmdb.ca) and METLIN (http://metlin.scripps.edu). The datasets were analyzed using MetaboAnalyst R [13]. The peak areas were normalized to internal standards. Metabolites with a percentage relative standard deviation (SD) of > 30% in the quality control samples were excluded, and the remaining data were log-transformed. Orthogonal partial least squares discriminant analysis (OPLS-DA) was used to determine metabolic profile differences among the groups. Important differential metabolites among the groups were defined based on a variable importance in projection value of > 1 and a false discovery rate of < 0.05. The Wilcoxon rank-sum test was used to compare the levels of the metabolites of interest among the groups.

#### Clinical variables and integrative analyses

The remaining analyses were performed using the R software. Categorical variables were expressed as numbers and frequencies. For continuous variables, normally distributed data were presented as means and SDs, and non-normally distributed data were shown as medians and interquartile ranges. To determine differences among the study groups, we used the χ^2^ test for categorical variables, unpaired Student’s *t*-test for normal quantitative variables, and the Mann–Whitney *U*-test for non-normal quantitative variables. Correlations among microbial, metabolomic, and clinical factors were calculated using Spearman’s correlation analysis. A co-abundance network analysis was performed using the NAMAP algorithm (*P* < 0.05, *r* > 0.7) using MetagenoNets [14]. The discriminatory markers for each profile type were determined using random forest (RF) models. For each test, the performance of the RF models for discriminating different CKD groups from the HC group was examined by receiver operating characteristic (ROC) curves constructed by pROC using 100-fold cross-validation. All multiple tests among the study groups were adjusted using the Benjamini–Hochberg method. Statistical significance was set at a *P*-value of < 0.05.

## Results

### Study population characteristics

We enrolled 68 patients with CKD (15 with mild CKD, 27 with moderate CKD, and 26 with ESRD) and 20 HCs [10, 11]. Table 1 and Additional Table 1 present the detailed characteristics. The CKD severity was positively associated with the serum creatinine level but negatively associated with eGFR and the serum albumin and hemoglobin (Hb) levels. Hypertension and diabetes were more prevalent in the ESRD and moderate CKD groups, respectively. Age, sex, body mass index, cause of CKD, and liver function did not differ between the groups.

**Table 1:** Clinical characteristics of study populations

Characteristics	Control (*n* = 20)	Mild-CKD (*n* = 15)	Mod-CKD (*n* = 27)	ESRD (*n* = 26)	*p*-value
Male, *n* (%)	7 (35.0)	7 (46.7)	8 (34.5)	12 (54.5)	0.143
Age (years), mean (SD)	56.1 (11.0)	53.0 (15.8)	60.4 (9.8)	59.8 (10.6)	0.365
BMI (kg/m^2^), mean (SD)	22.8 (2.1)	25.3 (2.5)	25.6 (4.4)	23.8 (3.9)	0.052
**Cause of CKD, *n* (%)**					0.735
Diabetes	—	1 (6.7)	5 (18.5)	8 (30.8)	
Hypertension	—	1 (6.7)	3 (11.1)	3 (11.5)	
Glomerulonephritis	—	4 (26.7)	6 (22.2)	5 (19.2)	
Nephrotic syndrome	—	3 (20.0)	5 (18.5)	3 (11.5)	
Polycystic kidney disease	—	1 (6.7)	2 (7.4)	2 (7.7)	
Others	—	5 (33.3)	6 (22.2)	5 (19.2)	
**Comorbidities, *n* (%)**					
Type 2 diabetes	—	1 (6.7)	8 (29.6)	12 (46.2)	0.024
Hypertension	—	3 (20.0)	19 (70.4)	20 (76.9)	< 0.001
**Laboratory index, median (IQR)**					
eGFR (ml/min/1.73 m^2^)	106.5 (98.2–117.0)	82.7 (67.8–107.1)	25.8 (19.0–40.3)	7.1 (4.9–9.6)	< 0.001
Serum creatinine (μmol/L)	57.5 (49.5–60.8)	87.5 (78.3–99.0)	181.0 (151.0–238.0)	593.0 (471.5–806.0)	< 0.001
WBC (× 10^12/^L)	6.3 (4.6–7.0)	5.9 (5.3–7.4)	6.7 (5.3–7.7)	5.9 (5.1–6.7)	0.408
Hemoglobin (g/L)	144.5(136.3–152.8)	141.5 (126.0–149.5)	111.0 (98.0–129.0)	88.5 (77.8–101.0)	< 0.001
AST (U/L)	15.0 (12.5–23.0)	14.5 (9.4–23.5)	12.1 (8.7–22.0)	17.8 (10.7–37.0)	0.286
ALT (U/L)	18.9 (16.5–22.3)	16.2 (15.2–22.3)	16.0 (15.5–20.5)	16.7 (11.0–23.8)	0.474
Albumin (g/L)	47.6 (47.6–51.6)	42.4 (33.8–45.5)	40.0 (36.2–43.2)	36.5 (32.6–40.8)	< 0.001
Triglycerides (mg/dL)	1.7 (1.3–2.2)	1.9 (1.3–2.6)	2.3 (1.6–3.3)	1.4 (1.0–2.5)	0.073
Total cholesterol (mg/dL)	4.6 (4.5–5.5)	4.8 (4.1–5.7)	4.3 (3.6–5.1)	4.2 (3.5–5.1)	0.209

Abbreviations: *CKD* chronic kidney disease, *mod-CKD* moderate CKD, *ESRD* end-stage renal disease, *eGFR* estimated glomerular filtration rate, *BMI* body mass index, *SD* standard deviation, *IQR* interquartile range, *AST* aspartate aminotransferase, *ALT* alanine aminotransferase

### Gut microbial alterations in patients with CKD

We performed metagenomic analyses in all CKD groups and HCs to explore the link between the gut microbiome and CKD severity. We detected relatively decreased *α*-diversity in the CKD groups using the Chao1 and Shannon indices (Fig. 1B–C and Additional Table 2). Furthermore, the PCoA based on Bray–Curtis distances indicated significantly different gut microbial structures among the four groups (*P* = 0.004; Fig. 1D).

We observed considerable differences in the gut microbial profiles between the CKD and HC groups from the phylum to genus levels. At the phylum level, the relative abundance of *Proteobacteria* was enriched in the ESRD group, whereas *Euryarchaeota* was more abundant in the HC group (Fig. 1E and Additional Table 2). At the phylum level, the relative abundance of *Proteobacteria* was enriched in the ESRD group, whereas *Euryarchaeota* was more abundant in the HC group. At the family level, *Veillonellaceae*, *Lactobacillaceae*, and *Eubacteriaceae* significantly decreased, but *Enterobacteriaceae* gradually increased in the ESRD group. Meanwhile, *Rikenellaceae*, *Lactobacillaceae*, and *Clostridiaceae* decreased in the moderate CKD group, and *Selenomonadaceae* increased in the mild CKD group (Fig. 1F and Additional Table 2). Notably, *Lactobacillaceae* and *Veillonellaceae* were positively correlated with eGFR as a CKD-severity index, whereas *Enterobacteriaceae* was negatively correlated (Additional Fig. 1A). At the genus level, *Roseburia*, *Faecalibacterium*, *Eubacterium rectale*, *Eubacterium*, and *Ruminococcus* considerably decreased in the moderate CKD and ESRD groups, whereas *Flavonifractor* and *Citrobacter* increased in these groups (Fig. 1G and Additional Table 2), consistent with the shifts reported in previous studies of microbial populations in patients with CKD [6, 8].

### CKD severity-related changes in microbial species compositions and functions

We observed two altered patterns of these species; the first pattern varied across the mild CKD to ESRD groups, whereas the second varied in only a specific CKD group (Fig. 2A). Specifically, the first pattern included 18 CKD microbial species; during CKD progression, the levels of four species increased (*Citrobacter freundii* and *Citrobacter werkmanii* [of the *Enterobacteriaceae* family belonging to the *Proteobacteria* phylum], *Flavonifractor plautii*, and *Anaerostipes caccae*) and those of 14 species decreased (*Methanobrevibacter smithii*, *Coprococcus comes*, *Coprococcus eutactus*, *Clostridium sporogenes*, *Ruminococcus callidus*, *Ruminococcus bromii*, *Roseburia hominis*, *Faecalibacterium prausnitzii*, *Veillonella parvula*, *Megasphaera elsdenii*, *Dialister succinatiphilus*, *Acidaminococcus intestini*, *Faecalicoccus pleomorphus*, and *Subdoligranulum variabile*, Fig. 2A). The second pattern included eight species. In the mild CKD group, the *Megasphaera micronuciformis* level increased. In the moderate CKD group, *Alistipes indistinctus*, *Alistipes inops*, and *Bacteroides uniformis* levels decreased (Fig. 2A). In the ESRD group, the *Turicibacter sanguinis* level increased, but the *Streptococcus mutans*, *Bifidobacterium adolescentis*, and *Lactobacillus crispatus* levels decreased (Fig. 2A). Furthermore, microbial species, such as *F. plautii*, *C. werkmanii*, *Ruminococcus* spp., *R. hominis*, *E. rectale*, *F. prausnitzii*, and *C. sporogenes*, were closely associated with eGFR and serum Alb and Hb levels (Additional Fig. 1B and Additional Table 3). Among all clinical factors, hypertension, diabetes, serum albumin, and serum Hb significantly differed among the CKD groups; therefore, they were included as confounders in the multivariate analyses. However, the CCA found no association between CKD severity-related gut microbiome and hypertension, diabetes, or the albumin and Hb levels (*P* = 0.077, Additional Fig. 1C). Moreover, the cause of CKD did not differ among the CKD groups. These results suggest that disease status is a major factor associated with gut microbiota.

**Fig. 3:**
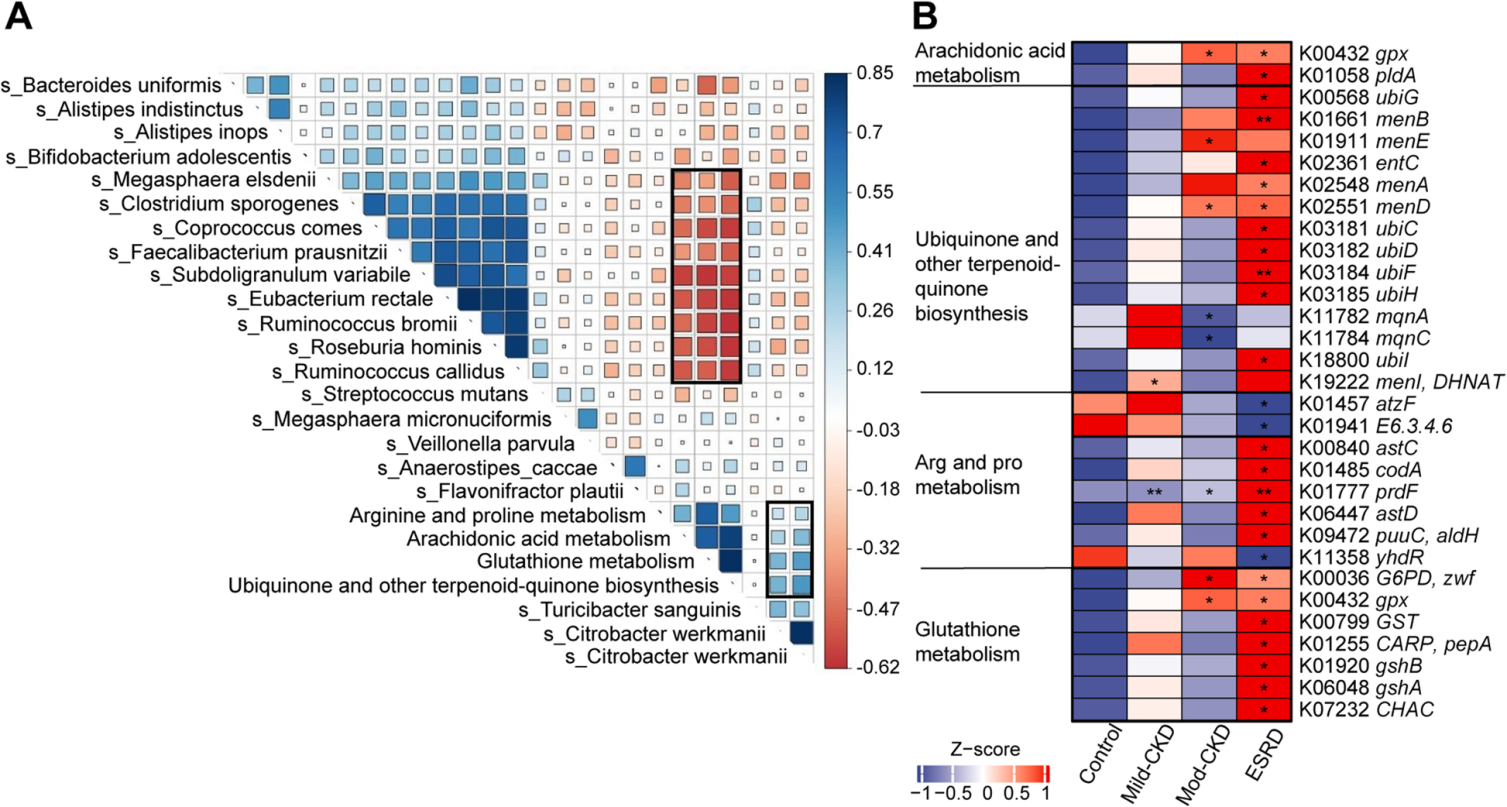
Correlations between the CKD severity-related microbial species and functions. **A** Heatmap representation of spearman correlation between CKD severity-related microbial species and corresponding KEGG pathways. **B** Relative abundance of KO genes involved in arginine and proline metabolism, arachidonic acid metabolism, ubiquinone and other terpenoid-quinone biosynthesis, and glutathione metabolism that showed significant differences in at least one of CKD groups compared to the HC were shown in the heatmap (*P<0.05, **P<0.01, Wilcoxon rank-sum test). mod-CKD, moderate CKD; ESRD, end-stage renal disease

We also identified 7778 KO genes associated with 69 altered functional pathways among the CKD severity groups. Based on the LEfSe analysis, the mild CKD group was characterized by the expression of microbial genes related to arginine and proline metabolism. The ESRD group was characterized by 10 KEGG pathways, half of which were involved in the inflammatory reaction and oxidative stress (Fig. 2B). Of note, the four essential metabolic pathways involved in arginine and proline, arachidonic acid (AA), and glutathione metabolism and the ubiquinone and other terpenoid-quinone biosynthesis pathways closely correlated with the specific microbial species that changed with CKD severity. As CKD progressed, 11 species had strong associations with the above four metabolic pathways; the levels of nine species decreased (*C comes*, *C. sporogenes*, *R. callidus*, *R. bromii*, *R. hominis*, *E. rectale*, *F. prausnitzii*, *M. elsdenii*, and *S. variabile*), and two species increased (*C. freundii* and *C. werkmanii*) (Fig. 3A).

**Fig. 4:**
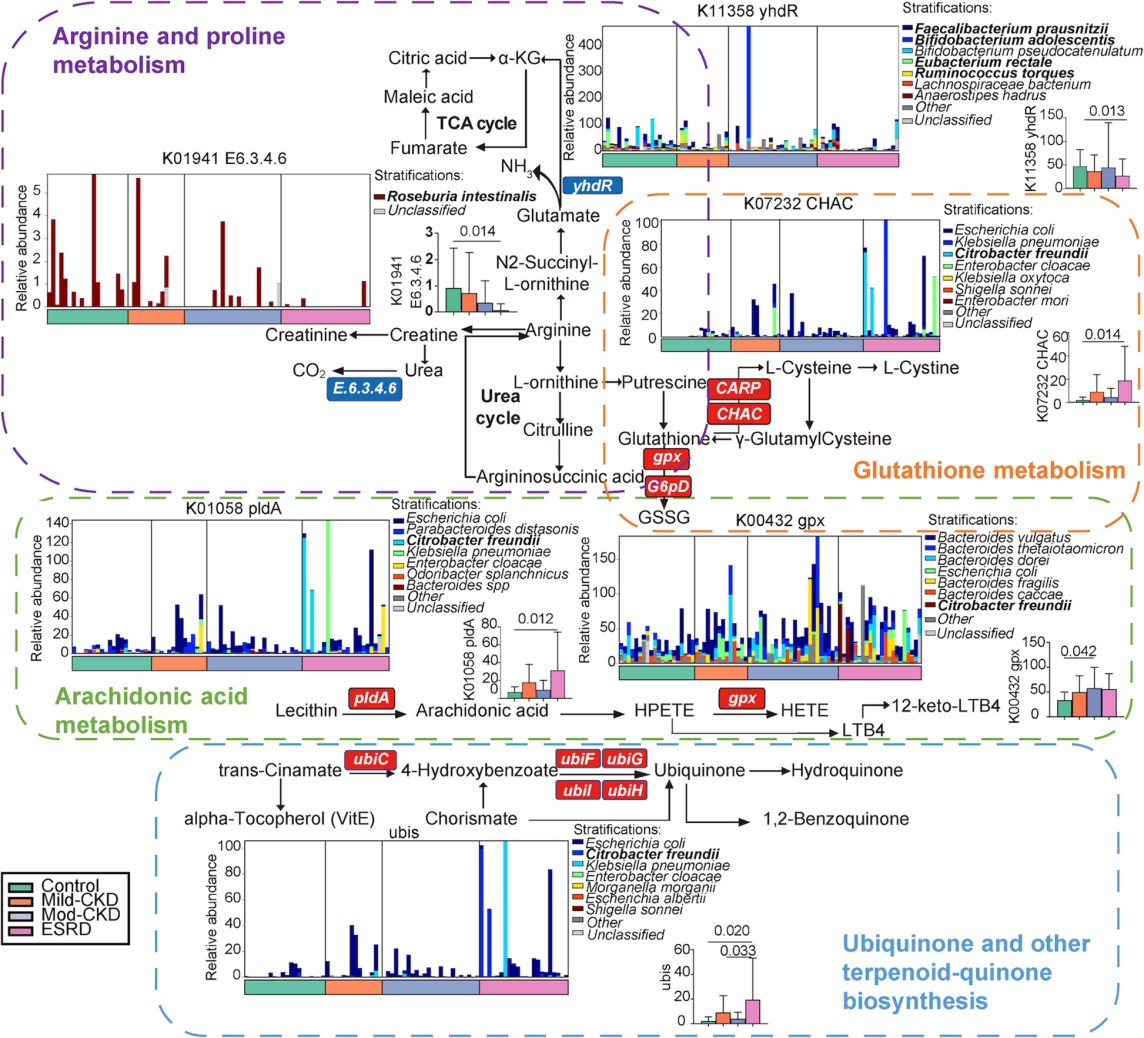
CKD severity-related changes of gut microbiome summarized in KO genes and KEGG pathways. Representative KO genes are shown in pathway modules modified from KEGG pathway maps “arginine and proline metabolism,” “arachidonic acid metabolism,” “ubiquinone and other terpenoid-quinone biosynthesis,” and “glutathione metabolism.” Except for the *ubis*, each box in a pathway represents a KO gene and is highlighted in red for elevation or in blue for depletion at any of CKD groups. The *ubis* included *ubiC*, *ubiF*, *ubiG*, *ubiH*, and *ubiI* genes. Bar plots show the relative abundance of KO genes and corresponding microorganism within each of the four groups, respectively. GSSG, glutathione disulfide; LTB4, leukotriene B4; α-KG, α-ketoglutaric acid; and TCA cycle, tricarboxylic acid cycle. mod-CKD, moderate CKD; ESRD, end-stage renal disease

Next, we investigated the roles of the microorganisms and their harbored genes in the four metabolic pathways essential in CKD. We identified significant differences in the corresponding KO genes between the CKD and HC groups (Fig. 3B and Additional Table 4). Representative reaction pathways were manually constructed by referring to the literature or modifying the KEGG pathway reference maps (Fig. 4) [12, 15]. Accordingly, AA and glutathione metabolism and ubiquinone and other terpenoid-quinone biosynthesis pathway disturbances were partly attributed to an increase in *C. freundii* and other *Enterobacteriaceae* species that contain specific enzymes for producing oxidizing substances. Accordingly, AA and glutathione metabolism and ubiquinone and other terpenoid-quinone biosynthesis pathway disturbances were partly attributed to an increase in *C. freundii* and other *Enterobacteriaceae* species that contain specific enzymes for producing oxidizing substances [16, 17]. Furthermore, the abundance of genes *K01941* and *ydhR*, which encode urea carboxylase and aspartate aminotransferase, respectively, decreased, which was related to disordered arginine and proline metabolism. Notably, *R. hominis* contributed the most to *K01941* changes, and *Ruminococcus* spp., *F. prausnitzii*, *B. adolescentis*, and *E. rectale* contributed to *ydhR* changes (Fig. 4). These findings suggest that the dysregulation of essential microbial metabolic processes contributes to CKD progression.

### Fecal and serum metabolomic profiles based on CKD severity

**Fig. 5:**
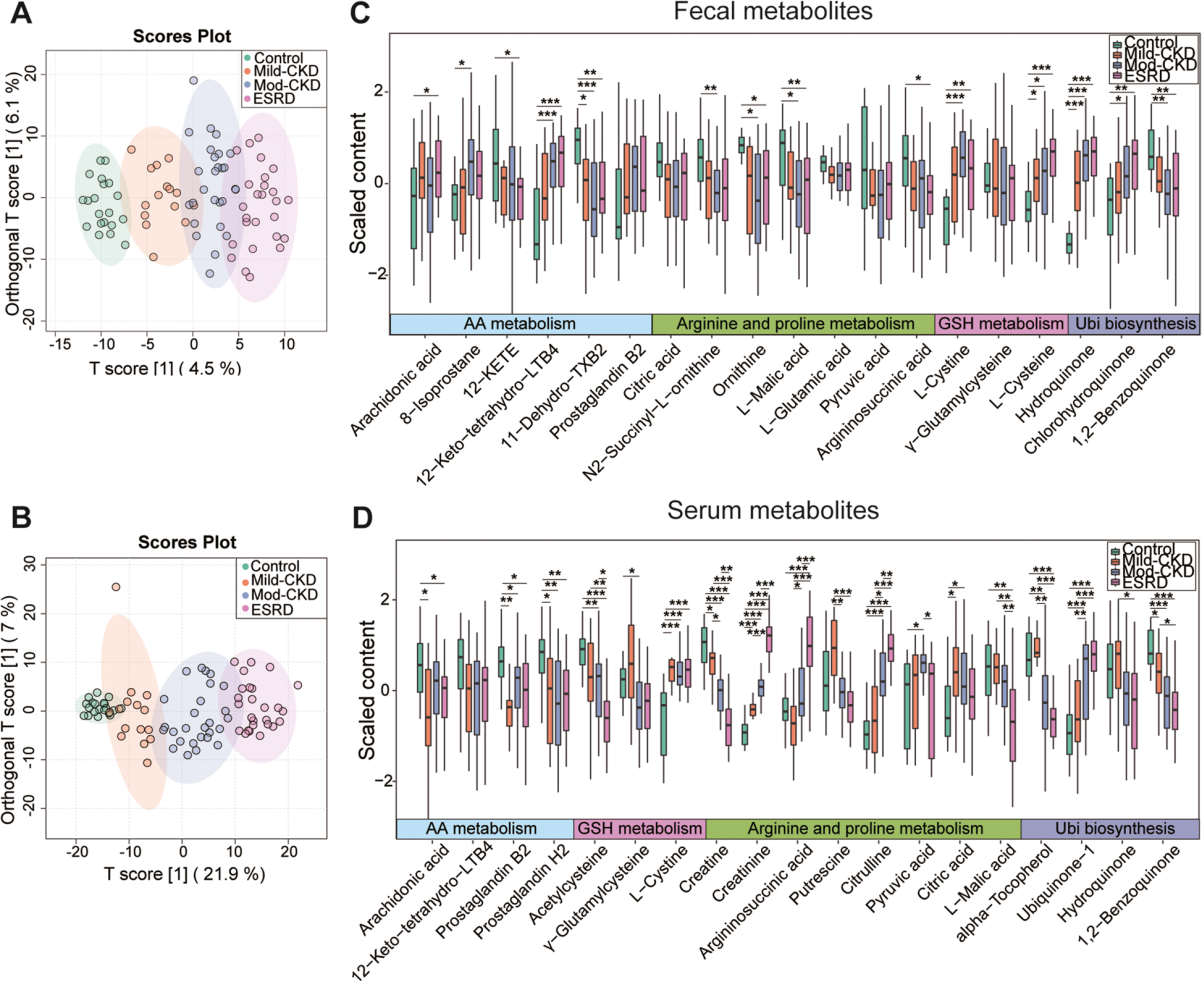
Alterations of fecal and serum metabolome across different CKD-severities. Orthogonal partial least squares discriminant analysis (OPLS-DA) of metabolites in the **A** fecal and **B** serum samples, with the ellipse represents the 95% confidence interval. Comparisons of **C** fecal and **D** serum metabolites related to arginine and proline metabolism, arachidonic acid metabolism, ubiquinone and other terpenoid-quinone biosynthesis, and glutathione metabolism showed significant elevation or depletion stratified by the severity of chronic kidney disease (CKD). Statistical analysis was performed by Wilcoxon rank-sum test (*P < 0.05, **P < 0.01, ***P < 0.001). Benjamini–Hochberg procedure was employed for the multiple test adjustments. GSH metabolism, glutathione metabolism; AA metabolism, Arachidonic acid metabolism; Ubi biosynthesis, ubiquinone and other terpenoid-quinone biosynthesis; mod-CKD, moderate CKD; ESRD, end-stage renal disease; LTB4, leukotriene B4; TXB2, thromboxane B2; 12-KETE, 12-keto-5,8,10,14-eicosatetraenoic acid

To define the functional alterations of CKD severity-related gut microbiota and their relationship with the host, we profiled the metabolome in fecal and serum samples using untargeted LC–MS/MS. Both score plots of OPLS-DA constructed on the fecal and serum metabolites clearly separated the HC group from the CKD groups (Fig. 5A–B). Furthermore, these separations became more apparent with CKD progression. In total, 103 of the 975 annotated fecal metabolites and 243 of the 642 annotated serum metabolites significantly differed between the CKD and HC groups (*VIP* > 1, *FDR* < 0.05, Additional Tables 5 and 6). Subsequent metabolomic analyses have confirmed the metabolites related to the four essential metabolic pathways during CKD progression.

Among the fecal metabolites, N2-succinyl-L-ornithine, ornithine, argininosuccinic acid, L-malic acid, 11-dehydro-thromboxane B2, 12-KETE, and 1,2-benzoquinone gradually decreased with increasing CKD severity, but the opposite was found for fecal 12-keto-tetrahydro-leukotriene B4 (12-keto-tetrahydro-LTB4), hydroquinone, chlorohydroquinone, L-cysteine, and L-cystine (Fig. 5C and Additional Table 7). We then assessed the relationships between these fecal metabolites and the clinical metadata. Strikingly, these CKD-related fecal metabolites significantly correlated with the CKD clinical parameters. For example, the levels of fecal hydroquinone, chlorohydroquinone, L-cysteine, L-cystine, and 12-keto-tetrahydro-LTB4 were inversely correlated with the eGFR and serum albumin and Hb levels. In contrast, metabolites enriched in the HC group, including L-malic acid, 11-dehydro-thromboxane B2, and 12-KETE, were positively correlated with eGFR and the serum albumin and Hb levels (Additional Fig. 2A and Additional Table 9). Interestingly, eGFR was closely associated with the L-cysteine (reductive form) to L-cystine (oxidized form) ratio (*r* = 0.299, *P* = 0.008), which is an anti-oxidation indicator [18].

Among the serum metabolites, the levels of creatine, creatinine, argininosuccinic acid, citrulline, L-cystine, and ubiquinone-1 were remarkably higher in the moderate CKD and ESRD groups than in the mild CKD or HC groups; the serum 1,2-benzoquinone, acetylcysteine, α-tocopherol, AA, prostaglandin H2, and prostaglandin B2 levels were the opposite (Fig. 5D and Additional Table 8). Furthermore, Spearman’s correlation analysis indicated that the CKD-related serum metabolites were tightly linked to the clinical indices of CKD. For example, serum creatine, acetylcysteine, α-tocopherol, and 1,2-benzoquinone were positively associated with eGFR and the serum albumin and Hb levels. Conversely, the serum creatinine, citrulline, L-cystine, argininosuccinic acid, and ubiquinone-1 levels were negatively correlated with the CKD clinical data (Additional Fig. 2B and Additional Table 9).

### Perturbed gut microbiota interactions with fecal and serum metabolism are related to CKD severity

**Fig. 6:**
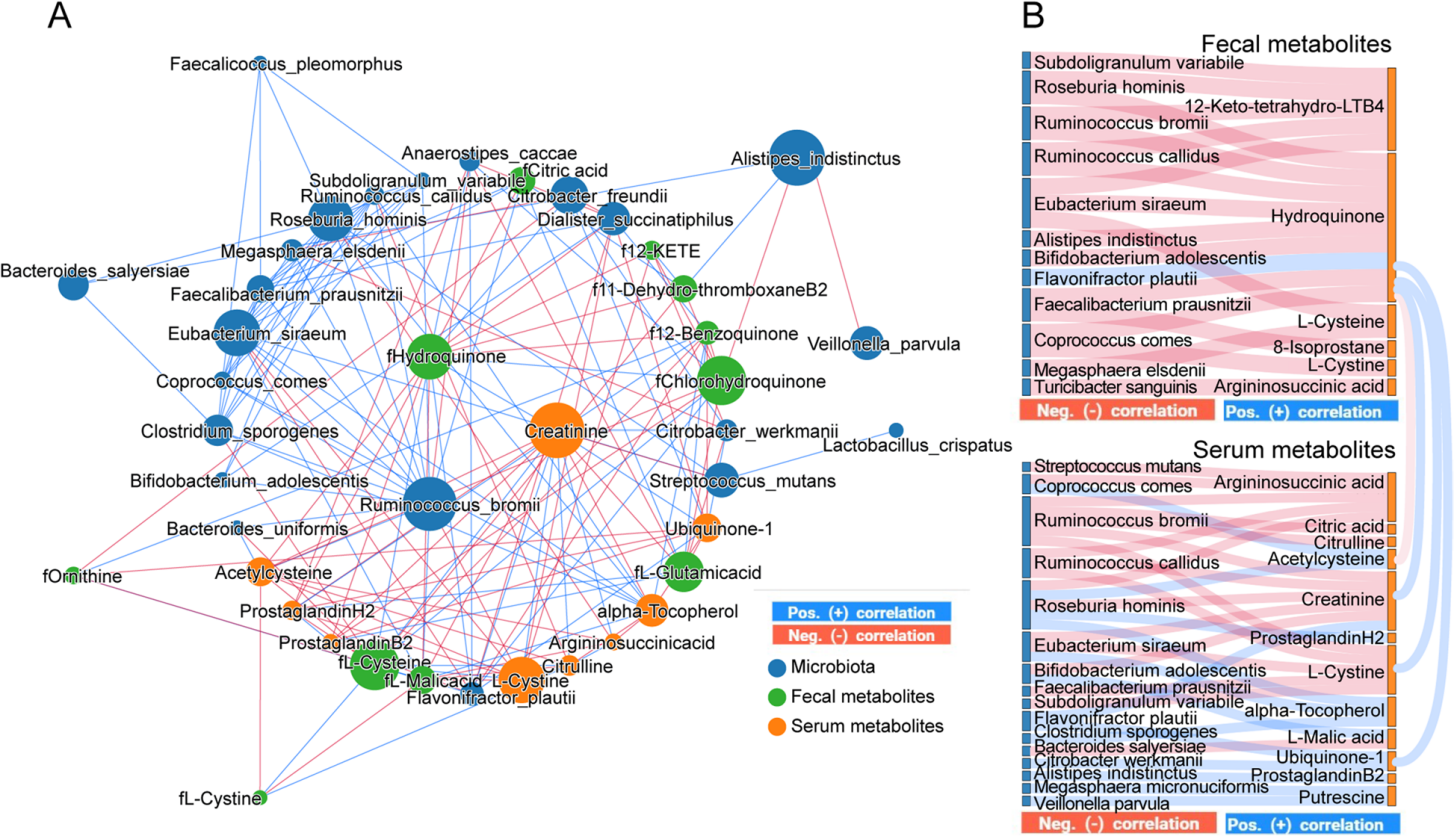
Interactions between gut microbiota composition and functional metabolites in feces and blood. **A** Integrated network of CKD severity-related microbial species, fecal metabolites, and serum metabolites. Networks were constructed using NAMAP with Spearman’s rank correlations between the gut microbiotas. The size of nodes represented the degree of corresponding factors. Direct correlations are indicated as blue edges and inverse correlations as red edges with a cutoff of *P* < 0.05 and *r* > 0.7. **B** The SANKEY diagrams show associations that are statistically significant with a cutoff of *P* < 0.05 and *r* > 0.7 based on bootstrapping of 150 iterations. The blue line and red line indicate positive and negative correlations, respectively. LTB4, leukotriene B4; 12-KETE, 12-keto-5,8,10,14-eicosatetraenoic acid

We performed an integrated network analysis based on metagenomic and metabolomic data to investigate the putative mechanisms between gut microbes and functional metabolites in disease progression (Fig. 6). Accordingly, we observed a complex co-occurrence pattern and a strong correlation between CKD severity-related bacteria and characterized metabolites from the four essential metabolic pathways. A striking characteristic was that *R. bromii*, fecal hydroquinone, and serum creatinine were identified as the main contributors to this integrated network, implying they have key roles in CKD progression. Interestingly, *R. bromii*, *R. callidus*, *R. hominis*, *Eubacterium siraeum*, *F. prausnitzii*, and *S. variabile* were well clustered and negatively correlated with the levels of fecal hydroquinone and serum creatinine; an opposite trend was observed for *F. plautii* (Fig. 6A). In addition, the *F. plautii*, *R. hominis*, *E. siraeum*, and *C. comes* counts were closely linked to fecal and serum metabolites from the ubiquinone and other terpenoid-quinone biosynthesis and glutathione metabolism pathways (Fig. 6B). Notably, fecal hydroquinone was positively correlated with serum creatinine, ubiquinone-1, and L-cystine but negatively associated with serum acetylcysteine. These findings confirm that gut microbiota dysbiosis, especially enriched levels of *F. plautii*, accompanied by depleted *R. bromii*, *R. callidus*, *F. prausnitzii*, and *R. hominis* levels may promote CKD progression by regulating the characterized metabolites involved in the four essential metabolic pathways between the gut and host.

### Noninvasive model discerns CKD severities

**Fig. 7:**
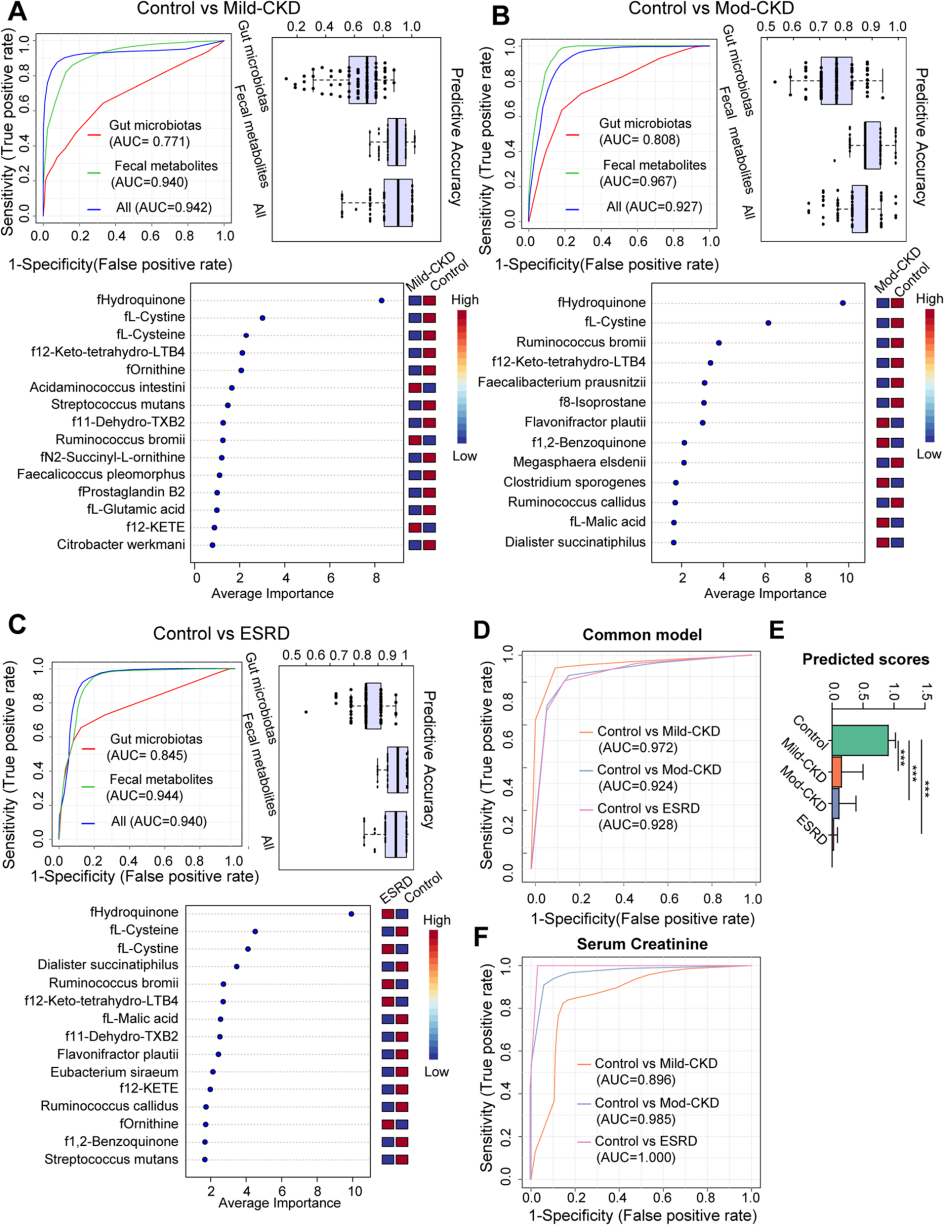
Diagnostic potential of gut microbiotas and fecal metabolites during multistep CKD progression. Gut metagenomic and metabolomic markers for noninvasively discriminating patients with **A** mild CKD, **B** moderate CKD, and **C** ESRD from the healthy controls (HC). These makers identified by random-forest (RF) classifiers based on species (red), or metabolites (green) alone, and the combination (blue) of the two features. Performance of RF classifiers was examined by receiver operating characteristic (ROC) curves and evaluated by 100-fold cross-validation. The box plots represent the average accuracy of models. **D** ROC analysis for the common model, taking into account fecal hydroquinone, L-cystine, 12-keto-tetrahydro-leukotriene B4, and *Ruminococcus bromii*, could correctly detect each of CKD groups from the HC. **E** Predicted score comparisons of the common model between the each of CKD groups and HC group using Mann–Whitney *U*-test (***P < 0.001). **F** ROC analysis for distinguishing each of CKD groups from HC group based on serum creatinine alone. mod-CKD, moderate CKD; ESRD, end-stage renal disease; LTB4, leukotriene B4; TXB2, thromboxane B2; 12-KETE, 12-keto-5,8,10,14-eicosatetraenoic acid

We constructed RF classifiers to identify different CKD severities and determine the potential of gut microbial and fecal metabolomic factors as noninvasive diagnostic markers (Fig. 7). We constructed three models based on the microbial species, fecal metabolites, or a combination (Fig. 7A–C). The models based on fecal metabolites with or without microbiota had greater performances than those based on gut microbiota alone (Fig. 7 A–C and Additional Table 10). This result verified that the overall functions of the microbiome outweigh the contribution of the individual microbial composition. In addition, a combination of four universal markers identified by RF, including fecal hydroquinone, L-cystine, 12-keto-tetrahydro-LTB4, and *R. bromii*, was used to construct a common model for discriminating the three CKD groups from HCs (Fig. 7A–C). This common model achieved a total area under the curve (AUC) of 0.972, 0.924, and 0.928 for patients with mild, moderate, and ESRD, respectively, after 100 cross-validations (Fig. 7D). The predicted score decreased as the CKD severity progressed (HC: mild CKD; moderate CKD: *ESRD* = 0.91: 0.16: 0.12: 0.032, *P* < 0.01, Fig. 7E). Interestingly, this generic model was superior to independent serum creatine for recognizing mild CKD (*AUC*: 0.972 vs. 0.896) (Fig. 7D–F), indicating that the gut microenvironment of patients with CKD may change earlier than the serum creatinine level.

## Discussion

Recent studies have reported that gut microbial dysbiosis is a key factor in CKD pathophysiology, making it a novel target for early diagnosis and personalized treatment [6, 19]. However, the functional potential of the gut microbiome of patients with CKD and its complex interplay with the host’s metabolism based on disease severity remains underestimated. Therefore, we performed a comprehensive study integrating multidimensional datasets of fecal metagenomics and fecal and serum metabolomes in a CKD cohort of varying disease severities. We identified gut microbiome and fecal and serum metabolome disruptions in patients with CKD, which may regulate toxic and oxidative stress metabolite disruptions to accelerate CKD progression. We also found that evaluating gut microbiota and fecal metabolites in combination might be a noninvasive biomarker for CKD diagnosis, especially in the early stages.

Gut microbiota disturbances occur in patients with CKD, which may contribute to renal dysfunction [6, 11]. Here, we identified a gut microbiome-derived signature comprising 26 microbial species that changed with CKD progression. Studies have reported a decreased abundance of short-chain fatty acid (SCFA)-producing bacteria, such as *Roseburia*, *Faecalibacterium*, and *Coprococcus*, in patients with CKD, which could promote CKD progression by impairing intestinal barrier function and stimulating excessive inflammation [4, 12, 20]. Consistent with previous studies, we observed a decrease in 14 SCFA-producing bacteria in the CKD group, including *R. bromii*, *R. callidus*, *R. hominis*, *E. rectale*, *F. prausnitzii*, *C. comes*, *C. eutactus*, *C. sporogenes*, *S. variabile*, *D. succinatiphilus*, *B. adolescentis*, *L. crispatus*, *A. indistinctus*, and *A. inops* [5, 7, 20].

Moreover, we found the abundances of four pathogenic bacteria changed as CKD progressed: *C. freundii*, *C. werkmanii*, *F. plautii*, and *A. caccae*. *C. freundii* and *C. werkmanii*, belonging to the *Enterobacteriaceae* family, are anaerobic bacteria that contain specific enzymes that produce oxidizing substances and pro-inflammatory uremic toxins [16, 17]. *F. plautii* may induce oxidative stress and low-grade systemic inflammation in its host. Furthermore, another study in a Chinese cohort found similar results to ours; *F. plautii* increased as the CKD severity increased [6, 12, 21]. Finally, enriched *A. caccae* levels have been observed in chronic inflammatory atopic dermatitis [22].

Functionally, the KEGG pathway analysis based on metagenomics revealed that gut dysbiosis in CKD was closely linked to disordered arginine and proline, AA, and glutathione metabolism and ubiquinone and other terpenoid-quinone biosynthesis pathways, which are involved in increased uremic toxins, inflammatory reactions, and oxidative stress [23–25]. Thus, these findings suggest that changes of the gut microbiotas during CKD progression were characterized by decreases of the 14 SCFA producers accompanied by increases of the four opportunistic bacteria, which may be attributed to the disbalanced function of oxidative stress and inflammation.

Metabolomic analyses of fecal and blood samples identified a distinct distribution of several metabolites from the four essential metabolic pathways in CKD. First, in arginine and proline metabolism, we found enriched urea cycle products in the serum of patients with CKD (e.g., creatinine, citrulline, and argininosuccinic acid). In contrast, the urea cycle metabolites were depleted in the fecal matter (e.g., N2-succinyl-L-ornithine, ornithine, and argininosuccinic acid). These factors may aggravate uremic toxic deposition during CKD progression. Second, AA metabolism involves a cluster of unsaturated fatty acids, which trigger aberrant inflammatory responses and oxidative stress [23]. Thus, enriched fecal 12-keto-tetrahydro-LTB4 and 12-KETE derived from AA metabolism may promote CKD progression by regulating oxidative stress and inflammation. Moreover, disturbances in glutathione homeostasis have been observed in CKD progression, which involves multiple pathologies under oxidative stress, such as cancer progression, neurodegenerative disorders, and cystic fibrosis [26]. Here, we found that the serum pro-oxidative L-cystine level increased, while the anti-oxidative acetylcysteine level decreased during CKD progression [18, 27, 28]. Interestingly, the fecal L-cysteine to L-cystine ratio, which serves as a redox sensor [18], gradually decreased with renal deterioration, indicating imbalanced metabolisms related to uremic toxins, and oxidative stress in the intestine and circulation play key roles in CKD progression.

Remarkably, this is the first report to highlight the influence of ubiquinone and other terpenoid-quinone biosynthesis pathways on CKD progression. The ubiquinone and other terpenoid-quinone biosynthesis pathways produce cofactors and vitamin E [29], which are oxidized derivatives of aromatic compounds originating from the chorismite of the shikimate pathway in bacteria [29]. Consistent with previous studies, we reported that the serum α-tocopherol level, also called vitamin E, decreased significantly during CKD progression [28]. This result may be because α-tocopherol provides effective chain breakage in lipid peroxidation to form *α*-tocopheroxyl radicals, resulting in the downregulation of α-tocopherol [28]. Our study further revealed that fecal hydroquinone, the most relevant fecal metabolite to CKD severity-related gut microbiota, was closely associated with the serum creatinine, L-cystine, and acetylcysteine levels. Thus, it could also serve as a universal marker for distinguishing CKD with different severities. These findings indicate an important role for fecal hydroquinone in CKD progression, but further investigation is required to identify the potential mechanisms.

**Fig. 8:**
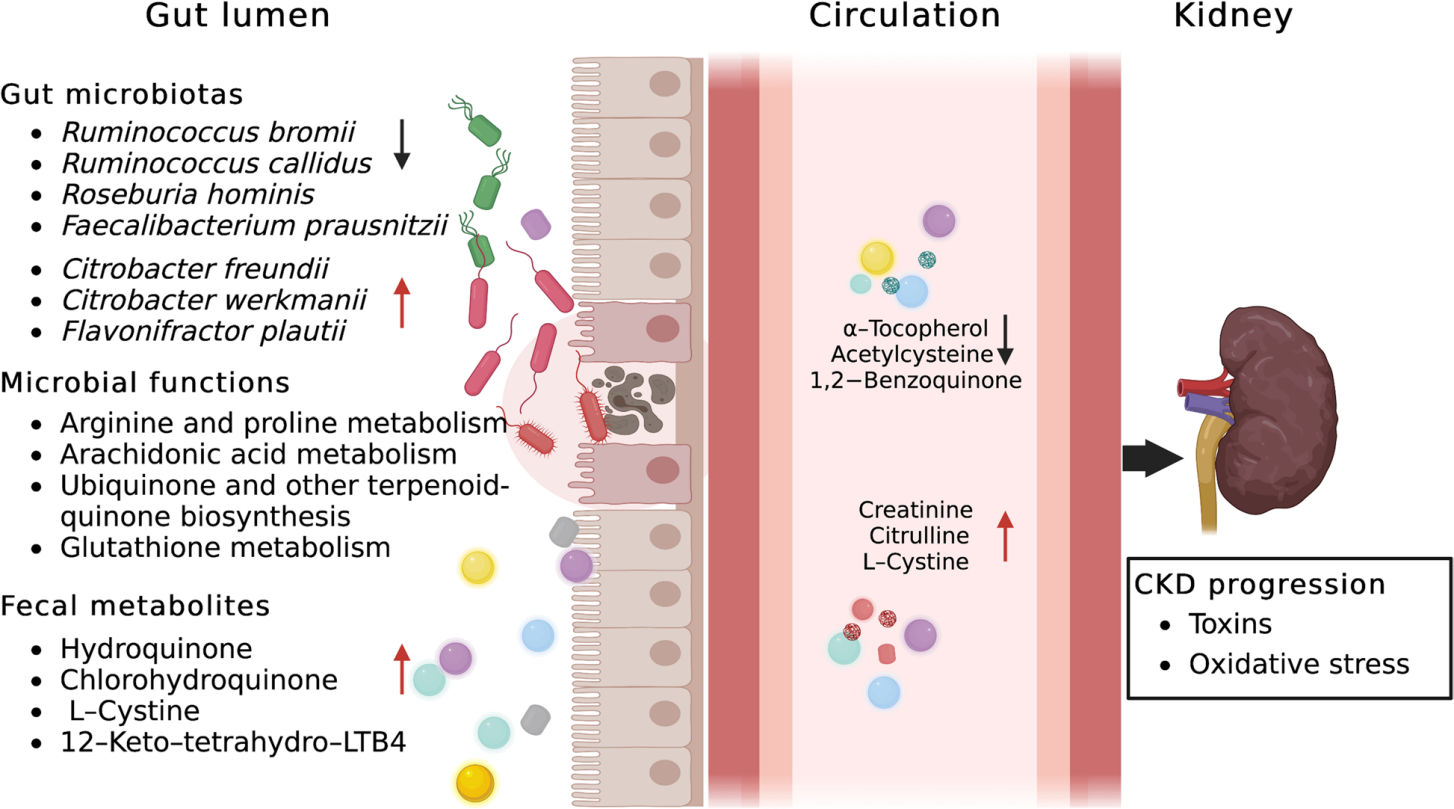
Proposed mechanism of perturbed gut microbiome together with fecal and serum metabolites in pathogenesis of CKD progression. Perturbation of CKD severity-related gut microbiotas may interact with disbalanced toxic and pro-oxidant metabolites from arginine and proline metabolism, arachidonic acid metabolism, ubiquinone and other terpenoid-quinone biosynthesis, and glutathione metabolism to accelerate disease progression. Red arrow indicates upregulation; black arrow indicates downregulation. LTB4, leukotriene B4

In addition, the integrated network analysis identified mutual interactions among the CKD-related species and metabolites from the four essential metabolic pathways. Consequently, we identified *R. bromii*, fecal hydroquinone, and serum creatinine as the primary contributors to this integrated network, indicating their key roles in CKD progression. *R. bromii*, a representative species of *Ruminococcaceae*, was one of the most significant taxa related to liver fibrosis severity in patients without obesity [30]. In the current study, the *R. bromii* abundance decreased as CKD severity increased, suggesting a therapeutic role in renal fibrosis and CKD progression. Moreover, *R. bromii*, *R. callidus*, *R. hominis*, and *F. prausnitzii* were inversely correlated with the fecal hydroquinone level, which was closely associated with serum creatinine, L-cystine, and acetylcysteine levels. The synthesis of vitamins and cofactors by intestinal bacteria influences the renal inflammatory oxidative stress status and sympathetic activity [7, 24]. Thus, we postulate that gut dysbiosis in CKD may disturb toxic and pro-oxidant metabolites directly or indirectly by regulating vitamin and cofactor metabolism, such as hydroquinone from ubiquinone and other terpenoid-quinone biosynthesis pathways, accelerating disease progression (Fig. 8). Among the new information obtained, reducing uremic toxins and inhibiting inflammatory oxidative stress through manipulation of gut microbiota seem to be a reasonable and novel therapeutic strategy to improve the survival of patients with CKD. However, further research is required to elucidate this potential mechanism.

Finally, our *R. bromii*, fecal hydroquinone, L-cystine, and 12-keto-tetrahydro-LTB4 model noninvasively detected CKD of differing severities. Notably, the power of this model is superior to that of the independent serum creatinine level for identifying mild CKD. This finding suggests that the breakdown of intestinal metabolism by gut microbiota appears earlier than serum uremia toxin storage in the host, providing an early diagnostic and therapeutic target for CKD.

Our study has some limitations. First, this was a cross-sectional study lacking dynamic patient follow-up. Therefore, a longitudinal study on the dynamic evolution of metagenomic and metabolomic profiles in CKD and the basic research underlying potential mechanisms are needed. Second, the presences of hypertension and diabetes significantly differed between the CKD groups in our study; these conditions have been reported to influence the gut microbiome [31, 32]. However, the CKD-related gut microbiome was not associated with the abovementioned clinical factors in CCA. Moreover, the cause of CKD did not differ among the CKD groups, suggesting that the disease status was the major contributor to CKD severity-related microbial profiles.

## Conclusions

We identified distinct changes in the gut microbiomes accompanied by functional alterations in arginine and proline, AA, and glutathione metabolism and ubiquinone and other terpenoid-quinone biosynthesis pathways during CKD progression. In addition, the intricate interactions among several CKD-related species and the metabolites from the four essential metabolic pathways were closely associated with CKD progression. Our multi-omics analyses identified potential mechanisms underlying the microbiota-gut-kidney axis in CKD progression and promising evidence for a novel target for early CKD diagnosis and intervention. Moreover, the disease severity-related changes in gut microbes combined with fecal metabolites provide a potential avenue for noninvasively diagnosing renal impairment, but this requires validation in a large population.

## Supplementary Information

**Additional file 1:** **Additional Tables 1–10.** Additional tables are separated in sheets in a single excel file. The first line in each sheet contains the title of each additional table. **Supplementary Table 1.** Post-hoc multiple analysis for study population characteristics. **Supplementary Table 2.** Comparisons of micaobial alpha diversity, beta diversity and taxa in each of the CKD groups with controls. **Supplementary Table 3.** Spearman correlation analysis between clinical data and CKD severity-specific species. **Supplementary Table 4**. Significant KO genes of those four metabolism pathways between each of the CKD groups and healthy controls. **Supplementary Table 5.** All differential fecal metabolites among each of the CKD groups and controls (VIP>1, FDR<0.05). **Supplementary Table 6.** All differential serum metabolites among each of the CKD groups and controls (VIP>1, FDR<0.05). **Supplementary Table 7.** Comparison of fecal metabolites related to Arginine and proline metabolism, Arachidonic acid metabolism, Ubiquinone and other terpenoid-quinone biosynthesis and Glutathione metabolism between each of the CKD groups and healthy control group.Statistical analysis was performed by Wilcoxon rank-sum test. Benjamini-Hochberg procedure was employed for the multiple test adjustments. **Supplementary Table 8.** Comparison of serum metabolites related to Arginine and proline metabolism, Arachidonic acid metabolism, Ubiquinone and other terpenoid-quinone biosynthesis and Glutathione metabolism between each of the CKD groups and healthy control group.Statistical analysis was performed by Wilcoxon rank-sum test. Benjamini-Hochberg procedure was employed for the multiple test adjustments. **Supplementary Table 9.** Spearman correlation analysis between clinical data and CKD severity-specific metabolites. **Supplementary Table10.** Performance of random-forest models discriminating CKD patients at different groups from controls based on 100-fold Cross-Validation.

**Additional file 2:** **Additional Fig. 1.** Correlation analysis between CKD severity-related microbiotas and clinical parameters. (**A**) Spearman correlation analysis showed significant relationships between eGFR and CKD severity-related microbial family, comprising *Enterobacteriaceae*, *Lactobacillaceae* and *Veillonellaceae* (*P*<0.05). (**B**) Heatmaps representation of spearman correlation between continuous clinical parameters and the CKD severity-related species (*P<0.05, **P<0.01, ***P<0.001). (**C**) Multivariate analysis by using canonical correspondence analysis (CCA) showed that there were insufficient influences of hypertension (HBP), type 2 diabetes (D2M), hemoglobin (HB) and serum albumin (Alb) on CKD severity-related microbial species.

**Additional file 3:** **Additional Fig. 2.** Association of clinical phenotypes and CKD severity-related metabolites. Heatmaps representation of spearman correlation between clinical parameters and the CKD severity-related metabolites of arginine and proline metabolism, arachidonic acid metabolism, ubiquinone and other terpenoid-quinone biosynthesis and glutathione metabolism in (**A**) fecal and (**B**) blood samples (*P<0.05, **P<0.01, ***P<0.001).

## Data Availability

The raw sequence data will be deposited and available in the NCBI database under accession number PRJNA800594.

## Abbreviations

AA: Arachidonic acid
Alb: Albumin
AUC: Area under the curve
ANOVA: One-way analysis of variance
BMI: Body mass index
CCA: Canonical correspondence analysis
CKD: Chronic kidney disease
CSS: Cumulative-sum scaling
ESRD: End-stage renal disease
eGFR: Estimated glomerular filtration rate
FDR: False discovery rate
FMT: Fecal microbiota transplantation
HB: Hemoglobin
HC: Healthy controls
HDL: High-density lipoprotein
IQR: Interquartile range
KEGG: Kyoto Encyclopedia of Genes and Genomes
KO: KEGG orthologs
LC-MS: Liquid chromatography-mass spectrometry
LEfSe: Linear discriminant analysis effect size
LTB4: Leukotriene B4
OPLS-DA: Orthogonal partial least squares discriminant analysis
PCoA: Principal coordinate analysis
PERMANOVA: Permutational multivariate analysis of variance
PERMDISP: Permutational analysis of multivariate dispersions
RF: Random forest
ROC: Receiver operating characteristic
SD: Standard deviation
SCFA: Short-chain fatty acid
TSS: Total sum normalization
TXB2: thromboxane
VIP: Variable importance
12-KETE: 12-keto-5,8,10,14-eicosatetraenoic acid
