# Supplementary figures and images for: Perturbed gut microbiome and fecal and serum metabolomes are associated with chronic kidney disease severity
Source: Microbiome. 2023 Jan 9;11:3. doi: 10.1186/s40168-022-01443-4 (PMC9827681; doi:10.1186/s40168-022-01443-4)

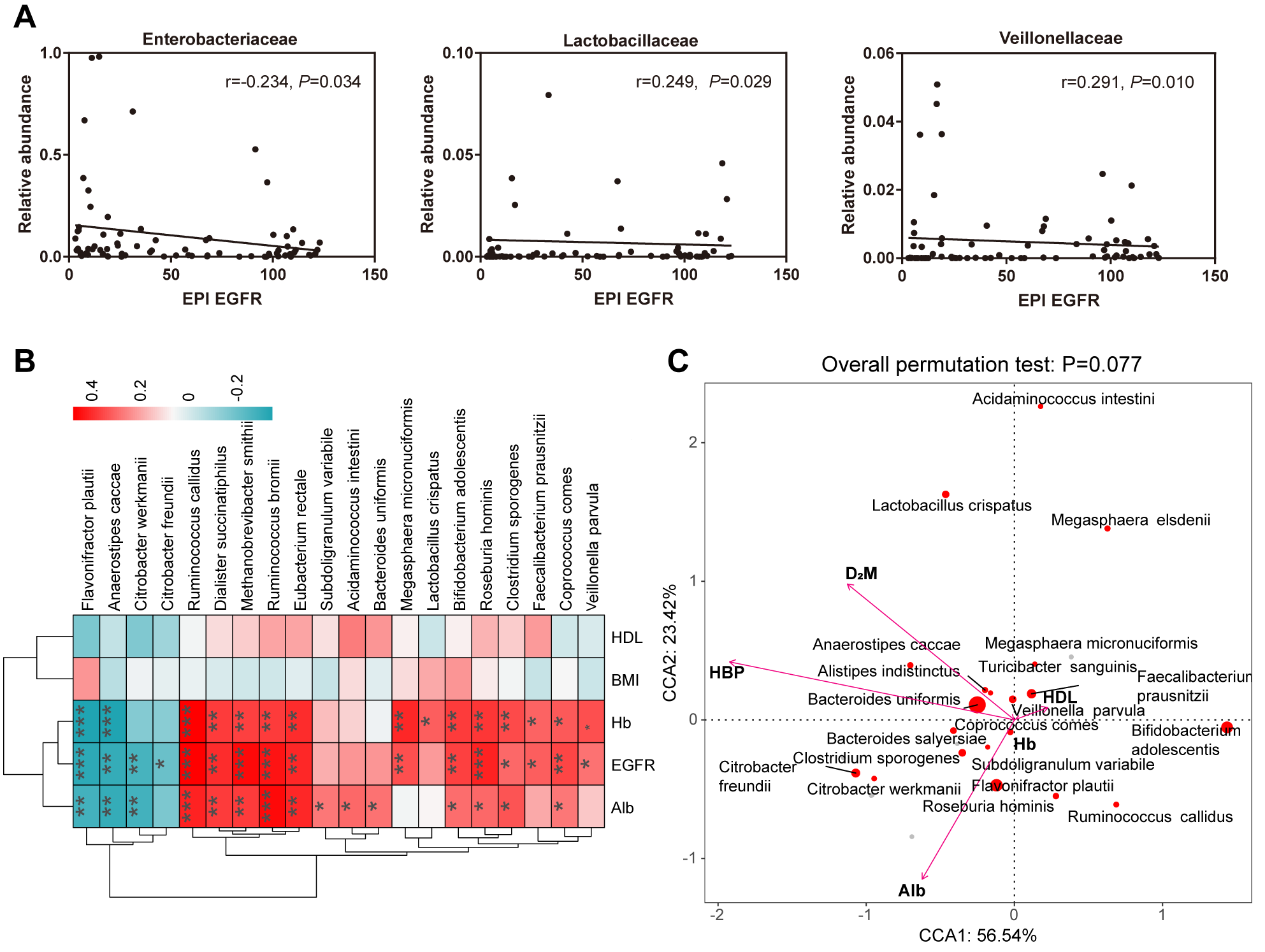

Supplement: Supplementary file 3 — Additional file 2: Additional Fig. 1. Correlation analysis between CKD severity-related microbiotas and clinical parameters. (A) Spearman correlation analysis showed significant relationships between eGFR and CKD severity-related microbial family, comprising Enterobacteriaceae, Lactobacillaceae and Veillonellaceae (P<0.05). (B) Heatmaps representation of spearman correlation between continuous clinical parameters and the CKD severity-related species (*P<0.05, **P<0.01, ***P<0.001). (C) Multivariate analysis by using canonical correspondence analysis (CCA) showed that there were insufficient influences of hypertension (HBP), type 2 diabetes (D2M), hemoglobin (HB) and serum albumin (Alb) on CKD severity-related microbial species. [file 40168_2022_1443_MOESM2_ESM.tif]

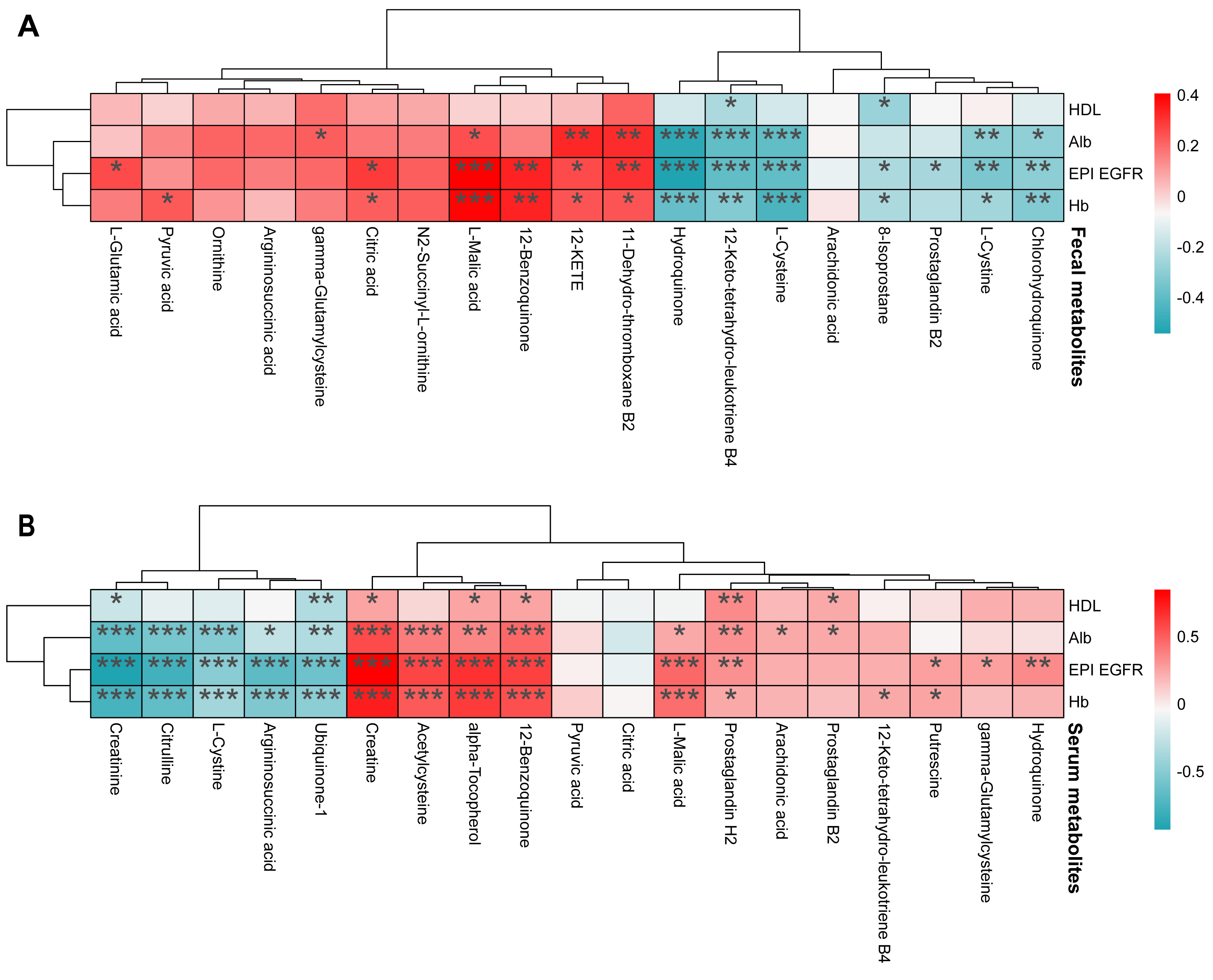

Supplement: Supplementary file 4 — Additional file 3: Additional Fig. 2. Association of clinical phenotypes and CKD severity-related metabolites. Heatmaps representation of spearman correlation between clinical parameters and the CKD severity-related metabolites of arginine and proline metabolism, arachidonic acid metabolism, ubiquinone and other terpenoid-quinone biosynthesis and glutathione metabolism in (A) fecal and (B) blood samples (*P<0.05, **P<0.01, ***P<0.001). [file 40168_2022_1443_MOESM3_ESM.tif]
